# Exploring the Path of Mediterranean Diet, Non-Alcoholic Fatty Liver Disease (NAFLD) and Inflammation towards 10-Year Cardiovascular Disease (CVD) Risk: The ATTICA Study 10-Year Follow-Up (2002–2012)

**DOI:** 10.3390/nu14122367

**Published:** 2022-06-07

**Authors:** Elena S. George, Ekavi N. Georgousopoulou, Duane D. Mellor, Christina Chrysohoou, Christos Pitsavos, Demosthenes B. Panagiotakos

**Affiliations:** 1 Institute for Physical Activity and Nutrition, School of Exercise and Nutrition Sciences, Deakin University, Geelong, VIC 2600, Australia; elena.george@deakin.edu.au; 2 Medical School, Australian National University, Canberra, ACT 2601, Australia; ekavi.georgousopoulou@nd.edu.au; 3 School of Medicine, University of Notre Dame Australia, Darlinghurst, SYD 2010, Australia; 4 Centre for Health and Medical Research, ACT Health Directorate, Canberra, ACT 2601, Australia; 5 Aston Medical School, Aston University, Birmingham B4 7ET, UK; d.mellor@aston.ac.uk; 6 First Cardiology Clinic, Athens Medical School, National Kapodistrian University of Athens, 10679 Athens, Greece; chrysohoou@usa.net (C.C.); greece.xlipidepart@internet.gr (C.P.); 7 Department of Nutrition and Dietetics, School of Health and Education, Harokopio University, 46 Paleon Polemiston St., Glyfada, Attica, 16674 Athens, Greece

**Keywords:** Mediterranean diet, non-alcoholic fatty liver disease, fatty liver index, inflammation, cardiovascular disease, cardiovascular risk

## Abstract

Background: Non-alcoholic fatty liver disease (NAFLD) is the leading cause of liver disease, affecting ~30% of the population and increasing CVD. This study aimed to explore the direct, indirect and combined effects of Mediterranean diet, NAFLD and inflammation on the 10-year CVD risk in a healthy adult population. Methods: Using baseline and 10-year follow-up data from the ATTICA study, adherence to Mediterranean diet was measured using MedDietScore, and presence of NAFLD at baseline was assessed using the fatty liver index (FLI). Participants’ 10-year CVD outcomes were recorded and C-reactive protein (CRP) was used as a surrogate marker for inflammation. The direct and indirect roles of these factors were explored using logistic regression models and the pathways between them were analysed using a structural equation model (SEM). Results: NAFLD prevalence was 22.9% and its presence was 17% less likely for every unit increase in MedDietScore. NAFLD presence at baseline was associated with increased 10-year CVD incidence (39.4% vs. 14.5%, *p* = 0.002), but when adjusted for MedDietScore, NAFLD was not an independent predictor of 10-year CVD risk. MedDietScore was an independent protective factor of 10-year CVD risk (OR = 0.989, 95% CI: 0.847, 0.935), when adjusted for NAFLD at baseline, age, gender, sedentary lifestyle and other confounders. Further exploration using SEM showed that MedDietScore was associated with CVD risk directly even when inflammation as CRP was introduced as a potential mediator. Conclusion: FLI as a proxy measure of NAFLD is a strong predictor of 10-year CVD risk, and this prognostic relationship seems to be moderated by the level of adherence to Mediterranean diet. Adherence to Mediterranean diet remained an independent and direct CVD risk factor irrespective of NAFLD status and CRP.

## 1. Introduction

Non-alcoholic fatty liver disease (NAFLD) is the most common liver disease worldwide and is present in 20–30% of populations in developed nations [1]. NAFLD is characterised by an accumulation of lipids involving ≥5% of hepatocytes in the absence of excessive alcohol intake [2]. Furthermore, NAFLD increases the risk of hepatocellular carcinoma and is an independent risk factor for cardiovascular disease (CVD) [3]. Rates of NAFLD are higher in individuals with type 2 diabetes (T2D) and obesity, affecting up to 70–90% of these individuals [4,5]. NAFLD is often considered the hepatic manifestation of the metabolic syndrome as it co-exists with other cardiovascular risk factors including hypertension, hyperlipidaemia, abdominal adiposity and insulin resistance [6,7]. The pathogenesis of NAFLD is largely driven by lipid accumulation associated with insulin resistance [8] and this is associated with chronic inflammation and oxidative stress impacting the long-term effects of NAFLD [9,10,11]. Evidence indicates that reduction in insulin resistance (IR) may reduce the onset of type 2 diabetes and atherosclerosis [12]. Therefore, management strategies should not only focus on reducing the synthesis and accumulation of lipids in hepatocytes [13], but also look to mitigate potential metabolic and inflammatory consequences. The reverse causality of this mechanism also needs to be considered with some experimental models suggesting inflammation potentially drives lipid accumulation [14]. 

Currently there is an absence of safe and effective pharmacotherapies to treat or manage NAFLD and the current recommended therapy is lifestyle intervention to elicit weight loss [15,16]. The optimal dietary pattern has not been explicitly identified and additional high-quality research is needed [17]. However, the limited literature to date suggests that a Mediterranean diet may be superior in the prevention and management of NAFLD [18]. It is hypothesised this plant-rich diet characterised by increased intake of vegetables, fruits and wholegrains, extra virgin olive oil and moderate fish, nuts, fermented dairy and limited amounts of red meat, has antioxidant and anti-inflammatory benefits which may ameliorate the pathogenesis of NAFLD [19]. To our knowledge, there are currently limited prospective studies that assess the role of dietary intake and, specifically, adherence to a Mediterranean diet and the long-term risk of individuals with NAFLD on CVD outcomes.

This study aimed to explore the direct, indirect and combined effects of adherence to a Mediterranean dietary pattern, NAFLD and inflammation levels in a healthy adult population on their 10-year risk of CVD. To address these aims, a cross-sectional analysis of the ATTICA study baseline data examined associations between adherence to Mediterranean diet and presence of NAFLD. In addition, the role of NAFLD status at baseline was explored as an independent risk factor for 10-year CVD risk using the 10-year follow-up data from the same study, using path analysis to explore interactions between Mediterranean diet, NAFLD and inflammation.

## 2. Materials and Methods

### 2.1. Sampling Procedure at Baseline Examination

The ATTICA study is a prospective population-based study conducted in the greater metropolitan area of Athens in Greece, including 78% urban and 22% rural population. The baseline examination of the study was carried out during 2001–2002 [20]. The sampling procedure anticipated enrolling only one participant per household; it was random, multistage and based on the age and gender distribution of the Attica region (census of 2001). Of the 4056 invited individuals at baseline examination, 3042 agreed to participate (75% participation rate); 1514 of the participants were men (18–87 years) and 1528 were female (18–89 years). All participants were interviewed and examined by trained personnel (cardiologists, general practitioners, dietitians and nurses) who used a standard questionnaire [20]. Exclusion of CVD at the baseline evaluation was performed through a detailed clinical evaluation by the physicians of the study. The examination was performed in the individuals’ homes or workplaces.

### 2.2. Baseline Measurements

#### Demographics and Lifestyle

The baseline evaluation included information about demographic characteristics (age, gender), personal and family history of hypertension, hypercholesterolemia and diabetes, family history of CVD, dietary and other lifestyle habits (i.e., smoking status and physical activity). In the present analysis, current smoking was defined as any use of tobacco or smoke during the last year. For the ascertainment of physical activity status, the international physical activity questionnaire was used (IPAQ) [21], as an index of weekly energy expenditure using frequency (times per week), duration (in minutes per time) and intensity of sports or other habits related to physical activity (in expended calories per time). Participants who did not report any physical activities were defined as physically inactive. Waist circumference was measured in the middle between the lowest rib and the iliac crest using an inelastic measuring tape to the nearest 0.5 cm, while waist to hip ratio was also calculated. Body mass index (BMI) was calculated as weight (in kilograms) divided by standing height (in meters squared). Obesity was defined as BMI greater than 29.9 kg/m^2^.

### 2.3. Dietary Assessment

Dietary intake was assessed using a validated semi-quantitative food frequency questionnaire (FFQ) [22], the EPIC-Greek questionnaire that was kindly provided by the Unit of Nutrition of Athens Medical School. This FFQ collected information for the whole year before the enrolment, including several seasonal foods as well [22]. Participants were asked if they had recently changed their diet, and whether they had received any interventions regarding their diet. Ethanol intake in a typical day was calculated using the same FFQ. The level of adherence to the Mediterranean diet was evaluated using an 11-item, composite index, the MedDietScore (range 0–55) [23]. In brief, for the consumption of foods presumed to be part of the Mediterranean pattern (i.e., those suggested on a daily basis or more than four servings per week, such as non-refined cereals, fruits, vegetables, legumes, olive oil, fish, and potatoes); lower scores are assigned when participants reported no, rare or moderate consumption, while higher scores were assigned when the consumption was according to the rationale of the Mediterranean pattern. For the consumption of foods presumed not to be part of the Mediterranean pattern (i.e., consumption of meat and meat products, poultry and full-fat dairy products), scores are assigned on a reverse scale. For alcohol, score 5 is assigned for consumption of less than 3 wine glasses per day, score 0 for consumption of more than 7 wineglasses per day and scores from 4 to 1 for consumption of 3, 4–5, 6 and 7 or 0 wineglasses per day, respectively. The theoretical range of the MedDietScore was 0–55 and higher values of this diet score indicate greater adherence to the Mediterranean diet. The accuracy and validity of the MedDietScore has been previously reported [24,25].

### 2.4. Clinical Assessment

Arterial blood pressure (3 recordings) was measured at the end of the baseline physical examination in a sitting position after resting for at least 30 min. Participants whose average blood pressure levels were greater or equal to 140/90 mmHg or who were using antihypertensive medication were classified as having hypertension. Blood samples were collected from the antecubital vein between 8 and 10 a.m., in a sitting position after 12 h of fasting and alcohol abstinence. Total serum cholesterol, HDL-cholesterol and triglycerides were measured using chromatographic enzymic method in a Technicon automatic analyser RA-1000 (Dade Behring, Marburg, Germany). Hypercholesterolemia was defined as total cholesterol levels greater than 200 mg/dL or the use of lipid-lowering agents. Blood glucose levels (mg/dL) measured with a Beckman Glucose Analyzer (Beckman Instruments, Fullerton, CA, USA). Diabetes mellitus (type 2) was defined according to the American Diabetes Association diagnostic criteria (i.e., blood glucose levels greater than 125 mg/dL classified participants as having diabetes). High-sensitivity CRP was assayed by particle-enhanced immunonephelometry (N Latex, Dade-Behring Marburg GmbH, Marburg, Germany) with a range from 0.175 to 1100 mg/dL. g-GT but also blood lipids were measured by using chromatographic enzymic method in an automatic analyser (RA-1000, Dade Behring, Marburg, Germany).

### 2.5. Definition of the Non-Alcoholic Fatty Liver Disease (NAFLD)

#### Fatty Liver Index

Fatty liver index (FLI), a non-invasive and useful surrogate marker for NAFLD in the general population (10), was employed in this study to identify the likely presence of NAFLD at baseline. The FLI produces a score between 0 and 100 and a cut off <30 (negative likelihood ratio = 0.2) was used to rule out the presence of NAFLD or ≥60 (positive likelihood ratio = 4.3) was used to deem the likely presence of hepatic steatosis [26]. The FLI was calculated using the following algorithm:

*FLI* = (*e* 0.953 × *loge* (*triglycerides*) + 0.139 × *BMI* + 0.718 × *loge* (*GGT*) + 0.053 × *waist circumference* − 15.745)/(1 + *e* 0.953 × *loge* (*triglycerides*) + 0.139 × *BMI* + 0.718 × *loge* (*GGT*) + 0.053 × *waist circumference* − 15.745) × 100 (1)

### 2.6. Follow-Up Examination (2011–2012)

During 2011-2012, the ATTICA study’s investigators performed the 10-year follow-up (mean follow-up time 8.41 years) [27]. All *n* = 3042 initially enrolled participants were contacted through telephone calls. Of those, there were *n* = 2583 who participated in the follow-up (85% participation rate) and investigators performed detailed evaluation of their medical records. Regarding CVD evaluation at follow-up, clinically accurate data were obtained from *n* = 2020 participants. Of the individuals that were lost to follow-up (i.e., *n* = 459), *n* = 224 were not found because of missing or wrong addresses and telephone numbers that they provided at baseline examination and *n* = 235 because they denied being re-examined. Mean age at baseline (±SD) was 45 ± 14 year for females and 46 ± 14 years for males (no difference with the overall baseline sample). Data were recorded on participants’: (a) vital status (death from any cause or due to CVD); (b) development of CHD (i.e., myocardial infarction, angina pectoris, other identified forms of ischemia—WHO-ICD coding 410–414.9, 427.2, 427.6–, heart failure of different types, and chronic arrhythmias—WHO-ICD coding 400.0–404.9, 427.0–427.5, 427.9–); (c) development of stroke (WHO-ICD coding 430-438).

### 2.7. Analytical Sample

**Figure 1:**
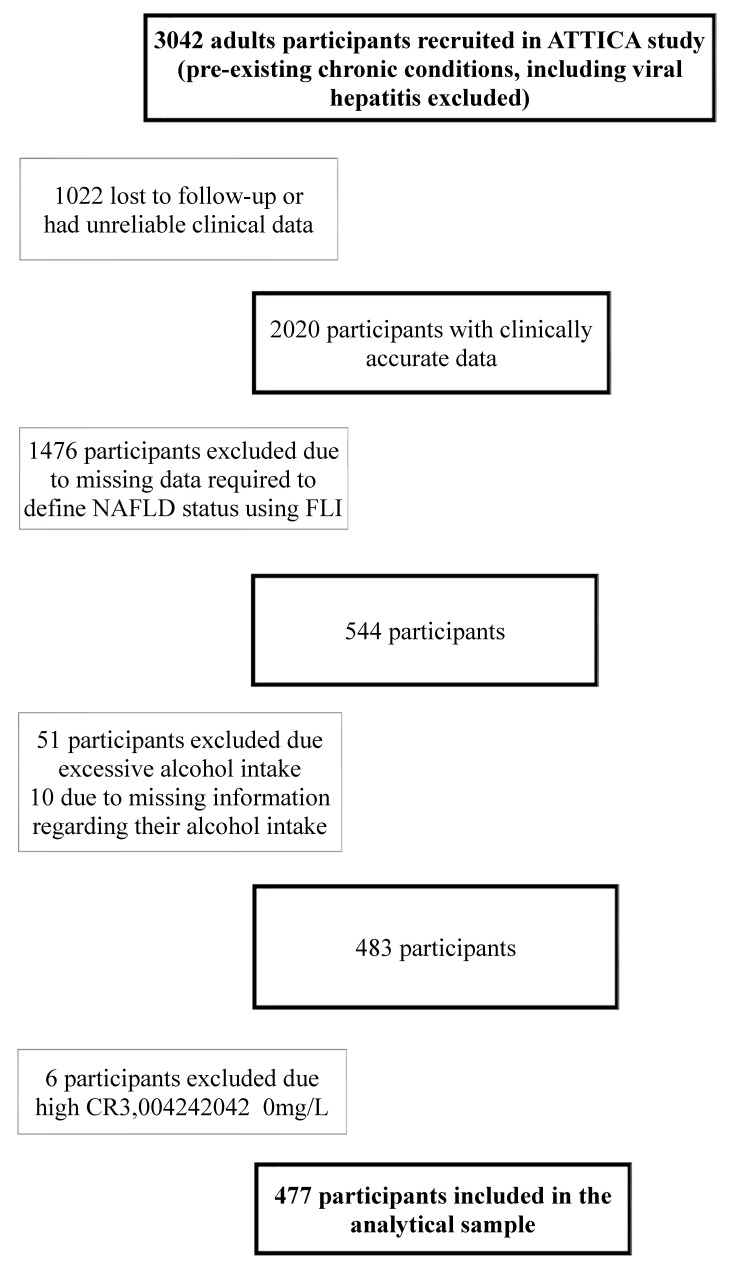
Flowchart describing exclusion reasons from ATTICA study to the analytical sample. NAFLD: Non-alcoholic fatty liver disease; CRP: C-Reactive Protein.

Participants that were either lost to follow-up or whose 10-year clinical data could not be validated were excluded from the analysis. With respect to participants with other known pre-existing causes of liver disease including viral hepatitis, they were excluded from the ATTICA study, alongside excluding participants with other chronic conditions. Participants with any missing data for any of the values required to calculate FLI (BMI, triglycerides, GGT, waist circumference) were excluded from the working sample. Based on NAFLD definition, excessive alcohol consumption was an exclusion criterion to ensure liver disease was non-alcoholic; thus, females consuming >20 g of ethanol/day and males consuming >30 g of ethanol/day were excluded from the analysis. Furthermore, participants with evidence of chronic inflammation including CRP levels above 10 mg/L were not included in the analytical sample (Figure 1). The analytical sample was compared to the overall sample of the ATTICA study and there were no important differences to suggest an introduction of reporting bias.

### 2.8. Bioethics

The study was approved by the Bioethics Committee of Athens Medical School and was carried out in accordance with the Declaration of Helsinki (1989) of the World Medical Association. Prior to the collection of any information, participants were informed about the aims and procedures of the study and provided their written signed consent.

### 2.9. Statistical Analysis

When applying the exclusion criteria, the working sample for the analysis was 544 individuals with complete data at baseline and 477 with complete 10-year follow-up. Variables were analysed on the basis of valid data without missing values imputations. Continuous variables were tested graphically for normality. Continuous variables were presented as mean values ± standard deviation (or median (1st, 3rd quartile) if not normally distributed) and categorical variables were presented as frequencies and relative frequencies. Associations between categorical variables were tested using the chi-square test, while between continuous variables using Pearson *r* or Spearman’s *rho* coefficients, for the normally and skewed variables, respectively. Comparisons between mean values of normally distributed variables for those who developed any CVD event and the rest of the participants were performed using Student’s *t*-test, after controlling for equality of variances using the Levene’s test. Comparisons of continuous variables that did not follow the normal distribution were performed using the non-parametric U-test proposed by Mann and Whitney. For the cross-sectional analysis, the odds ratios (OR) for the association between adherence to Mediterranean diet and presence of NAFLD were derived from binary logistic regression models. For the longitudinal analysis, the relative risk and the corresponding 95% confidence intervals (CI) of developing a CVD event during the 10-year period, according to the participants’ baseline characteristics, were estimated using binary logistic regression models. Confounding factors (i.e., gender, age, CRP, smoking status, sedentary lifestyle and education level) were included in multivariable models based on their associations in previous literature [28,29] and after examining associations between potential confounders, diet and outcome. Interaction terms between age groups and gender with MedDietScore were tested in the models, due to previous literature suggesting higher adherence to Mediterranean diet among females and older individuals [30]. Path analysis, using structural equation modelling (SEM) with observed data, was based on the aforementioned hypothesis in order to explore the paths by which diet affects CVD risk [27]. Specifically, a structural model was estimated in order to examine the direct and indirect role of adherence to Mediterranean diet and fatty liver index on 10-year CVD risk, taking into account CRP levels. The results of path analysis are presented as regression coefficients. All reported *p*-values are based on two-sided tests. SPSS version 19 (Statistical Package for Social Sciences, SPSS Inc., Chicago, IL, USA) and STATA version 15.0 were used for statistical analyses. Significance level was set at 5% and all reported *p*-values are based on two-sided tests and the corresponding 95% confidence interval (CI). SPSS version 21 (Statistical Package for Social Sciences, SPSS Inc, Chicago, IL, U.S.A.) software was used for all the statistical calculations.

## 3. Results

### 3.1. Cross-Sectional Analysis

Based on the FLI definition, NAFLD prevalence in the study sample was 22.9% and more frequent among males compared to females (44.1% vs. 14.4%, *p* < 0.001). The mean age of NAFLD-free individuals was significantly lower than subjects with NAFLD (47 ± 14.5 years vs. 53 ± 14.5 years, *p* < 0.001). The baseline characteristics of the participants with respect to the FLI groups are presented in Table 1. No differences were detected among groups for sedentary lifestyle, smoking habits and LDL-cholesterol levels (all *p*-values > 0.20).

Cross-sectional determinants of NAFLD presence were explored in a logistic regression multivariable model, presented in Table 2. Higher level of adherence to Mediterranean diet, as measured with MedDietScore, was associated with NAFLD with every unit increase in MedDietScore associated with 17% less likelihood of having NAFLD. Male gender was related with a five-fold likelihood of NAFLD presence, whilst every unit increase in CRP increased the odds of NAFLD presence by 28%. Age, educational level, sedentary lifestyle and smoking habits were not independently associated with NAFLD.

### 3.2. Prospective Analysis

NAFLD presence at baseline was associated with increased 10-year CVD incidence (39.4% vs. 14.5%, *p* = 0.002) in unadjusted analysis. More specifically, NAFLD risk groups were significantly associated with CVD incidence (*p* < 0.001), with the high-risk group reporting 29.2% CVD rate and the low-risk group reporting 8.8% CVD rate. Considering the cross-sectional association between adherence to Mediterranean diet and NAFLD, further analysis was performed to explore the long-term effect of NAFLD and Mediterranean diet on 10-year CVD risk, when taken into account simultaneously. As presented in Table 3, NAFLD and adherence to Mediterranean diet were separately and simultaneously associated with 10-year CVD risk. NAFLD was a very strong CVD risk predictor in a univariable model (OR = 2.4, 95% CI: 1.38, 4.29), but the association was no longer significant when adjusted for MedDietScore. Increased MedDietScore was a protective factor against 10-year CVD risk in univariable analysis (OR = 0.882, 95% CI: 0.843, 0.923), when adjusted for NAFLD at baseline (OR = 0.989, 95% CI: 0.847, 0.935) and in the fully adjusted model for age, gender, sedentary lifestyle, CRP levels, smoking status and education level (OR = 0.873 = 95% CI: 0.795, 0.959). A significant interaction term was found between age tertiles and MedDietScore (*p* = 0.001), which was also taken into account in the final model.

**Figure 2:**
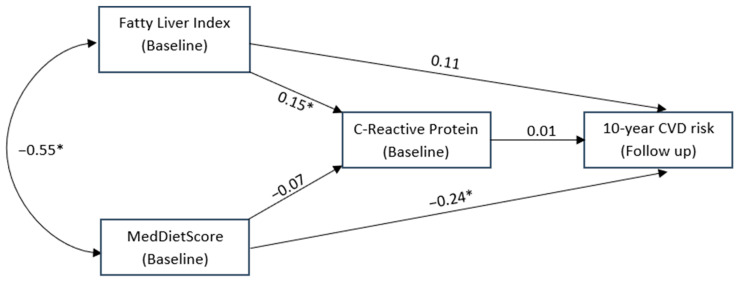
Structural equation model conceptualising the potential mediating role of C-Reactive protein levels on the association between fatty liver index, MedDietScore at baseline and the 10-year CVD risk. * is indicated where the relationship was statistically significant.

As shown in Figure 2, adherence to the Mediterranean diet as measured via MedDietScore was inversely associated with FLI. MedDietScore had a significant role on CVD risk that was independent and direct, whilst FLI was not directly associated with 10-year CVD risk in the same conceptual model but was associated with lower CRP levels. CRP levels were not directly associated with 10-year CVD risk and were not affected by MedDietScore. 

## 4. Discussion

The present analysis suggests that higher adherence to Mediterranean diet (assessed via MedDietScore) is associated with lower likelihood for NAFLD presence at baseline (assessed using FLI). Additionally, the presence of NAFLD at baseline showed predictive functionality for cardiovascular events (CVD) in a 10-year follow up of a Greek cohort. However, when both adherence to Mediterranean dietary pattern and presence of NAFLD at baseline were taken into account, NAFLD lost its predictive utility for 10-year CVD risk, whilst Mediterranean diet remained significantly protective. This could suggest that adherence to a Mediterranean dietary pattern is associated with reduced odds of co-existing with NAFLD and may ameliorate the negative effects of NAFLD on 10-year CVD risk. It was hypothesised that the potential mechanism driving any mediating effects of the Mediterranean diet on NAFLD and subsequently CVD risk was inflammation. This study showed through a structural equation model that, although there was an association between Mediterranean diet and NAFLD, as well as NAFLD and inflammation (using CRP), Mediterranean diet appeared to negate the effect of NAFLD on 10-year CVD risk.

The positive role of Mediterranean dietary pattern on CVD risk has been well defined [31], and more recently its potential to prevent and manage the growing epidemic of NAFLD has been identified [32], with the European Liver Association including the Mediterranean diet as the recommended diet for management of NAFLD [18]. The Mediterranean dietary pattern and its association with longevity is also well established [33] and evidence from prospective cohorts highlighting the inverse relationship between a Mediterranean dietary pattern and risk of CVD continues to grow [34]. It is thought that insulin resistance is one of the driving factors exacerbating type 2 diabetes and CVD and there is evidence to suggest that Mediterranean diet may moderate this effect [12]. An understanding of the role of the Mediterranean diet and health has been facilitated by the development of a number of dietary indices and scoring systems [35], some of which have been effectively applied to dietary intakes in countries outside of the Mediterranean basin [36]. The proposed pathway describing how a Mediterranean dietary pattern might reduce the risk of CVD has postulated a positive effect on reducing inflammation [31,37]. This could also explain its potential effects seen in reducing the risk of NAFLD, as well as being of potential therapeutic benefit in managing NAFLD. In clinical case reviews, individuals diagnosed with NAFLD have been shown to have a lower adherence to a Mediterranean diet [38], and this has been supported by data suggesting the potential of a Mediterranean dietary pattern to moderate NAFLD [19,39].

Despite the apparent connections between a Mediterranean dietary pattern, CVD risk and NAFLD, this pathway has yet to be investigated in a longitudinal prospective cohort. Consistent with previous research, data from this analysis of the ATTICA cohort demonstrated an association between Mediterranean diet score and CVD outcome [27], and a potential role of inflammation explaining the pathology and potential moderation by dietary factors. However, the analysis herein shows that NAFLD, a known independent risk factor for CVD [40], was less predictive for CVD when adherence to the Mediterranean diet was accounted for. This suggests that a Mediterranean dietary pattern has the potential to moderate the impact of NAFLD on CVD risk. It is worthy to note that although adherence to a Mediterranean dietary pattern is inversely associated with the presence of NAFLD in this cohort, there was a positive association between inflammatory marker (CRP) and NAFLD. This appeared to offer a consistent pathway linking the pathogenesis of NAFLD to increased risk of CVD risk factors and outcomes. However, this was not confirmed by the structural equation model. Although, only CRP was used as a surrogate for inflammation; thus, inclusion of alternative or several inflammatory markers may be a more robust way to further investigate this hypothesis. An alternative mechanism to explain these results may be that insulin resistance exacerbates de novo lipogenesis, which manifests via inflammation and underpins the pathological process driving NAFLD [41]. This is supported by the literature indicating that NAFLD and IR are correlated and exacerbate the development of pre-diabetes and diabetes [42,43].

As well as predicting CVD risk, the effect of FLI on CVD appears to be moderated by increasing MedDietScore. This is supported by underlying biological and mechanistic data, which suggest a diet rich in vegetable and fruit matter, including extra virgin olive oil rich in bioactive compounds including polyphenols, is cardioprotective. Both in vitro and in vivo laboratory studies have demonstrated potential mechanisms by which these compounds can moderate the inflammatory effects of fatty liver [44,45]. A systematic review of randomised controlled trials highlighted the importance of polyphenol-rich food products and cardiovascular outcomes [45]. Adherence to Mediterranean diet has also been associated with other cardiovascular risk factors such as lower central obesity, MetS and low-grade inflammation, consistent with the results demonstrated in this analysis [46]. Mediterranean diet has been associated with a more favourable biochemical profile in metabolic diseases and has also been suggested as protective against type 2 diabetes which has an established link with NAFLD [47,48]. Although this analysis using structural equation modelling could not demonstrate this pathway via CRP, it could be that CRP lacks sensitivity as a marker of inflammation associated with insulin resistance and fatty liver. Furthermore, CRP is susceptible to change with acute illness. However, it should be noted that the Mediterranean diet, especially in Mediterranean populations, may be a surrogate marker for an overall Mediterranean lifestyle including physical activity, social connectedness and less mental health stressors [49]. Thus, the present findings need to be examined in non-Mediterranean populations for further understanding of these associations.

### Strengths and Limitations

The presented study has numerous strengths and several limitations that should be considered when interpreting the results. To our knowledge, as well as reporting the association between Mediterranean diet and NAFLD cross-sectionally, this is the first original article to expand the exploration of the role of NAFLD in a prospective database with a 10-year follow-up. 

All exposures were measured at baseline and thus could have changed within the study follow-up, and these changes could not be accounted for in the analysis. However, this is common methodology in observational epidemiological studies and the findings are comparable with the majority of them, although well-established in epidemiological studies [50]. The use of the FLI as a proxy of NAFLD presence needs to be considered [26]; however, more accurate measures of NAFLD such as biopsy, ultrasound and magnetic resonance spectroscopy are either invasive or costly and not readily available in population studies. Nevertheless, FLI uses routinely assessed clinical measurements of BMI, triglycerides, GGT and waist circumference and therefore has application and translation to clinical practice. The utility of FLI needs to be further explored with respect to lifestyle interventions and how they moderate risk of CVD. When assessing metabolic risk using waist circumference, most definitions and ranges differentiate between genders and ethnicity; however, the ATTICA study is representative of the Greek population which is not ethnically diverse. Moreover, the study included male and females and different cut-off points have been applied as necessary. The rates of NAFLD within this study which reflect markedly higher rates in males are not consistent with global data for NAFLD; however, based on anthropometric data within Greece, higher rates of waist circumference are expected in males [51] and this is a parameter within the FLI. Despite the above findings, the diabetes prevalence was overall low in the study, but was consistent with the country’s official diabetes statistics [52]. Therefore, these results may be generalizable to Greece but not to other populations. 

## 5. Conclusions

Fatty liver index as a proxy measure of NAFLD is a strong predictor of CVD risk, but this prognostic relationship seems to be moderated by the level of adherence to the Mediterranean diet within a structural equation model. Further research is needed to examine the underlying mechanisms, with an emphasis on inflammation using additional inflammatory markers. This will assist in further understanding the role of the Mediterranean diet in NAFLD prevention and management.

## Figures and Tables

**Table 1:** Socio-demographic, lifestyle and clinical characteristics of the participants in respect to fatty liver index (FLI) groups at baseline (FLI: <30, 30–60, ≥60) *(n =* 544*)* ^1^.

Baseline Characteristics	FLI < 30 (*n* = 271)	30 ≤ FLI < 60 (*n* = 137)	FLI ≥ 60 (*n* = 136)	*p*-Value
Gender, male, *n* (%)	40 (21.9) *	61 (44.5)	82 (44.8)	<0.001
Age (years)	43.8 (13.5) *	54.6 (14.0)	53.5 (13.7)	<0.001
Education (school years)	12.6 (3.8) *	10.9 (4.5)	11.1 (4.3)	<0.001
MedDietScore (0–55)	27.5 (2.9) *	23.8 (5.0)	21.8 (5.0) *	<0.001
Ethanol intake (g/day) ^	0 (0, 0)	0 (0, 0)	0 (0, 20) *	<0.001
Sedentary lifestyle, yes, *n* (%)	176 (64.9)	83 (60.6)	96 (70.6)	0.219
Smoking, yes, *n* (%)	127 (46.9)	70 (51.1)	61 (44.9)	0.567
Body mass index (kg/m^2^)	23.1 (2.5) *	27.3 (2.3)	31.5 (4.1) *	<0.001
Waist circumference (cm)	78.7 (9.1)	93.0 (6.3)	105 (8.4)	<0.001
Diabetes mellitus, yes, *n* (%)	7 (2.6)	10 (7.3)	23 (16.9)	<0.001
Hypertension, yes, *n* (%)	48 (18.5)	44 (34.1)	67 (51.1)	<0.001
Hypercholesterolemia, yes, *n* (%)	111 (41.0)	83 (60.6)	72 (25.0)	<0.001
Triglycerides (mg/dL)	80.0 (59.0, 103) *	118 (92.0, 158)	151 (106, 228) *	<0.001 †
C-reactive protein (mg/L)	0.56 (0.26, 1.51) *	1.17 (0.62, 2.41)	1.81 (0.99, 3.59) *	<0.001 †
gamma-GT (IU/L)	14.0 (12.0, 17.0)	19.0 (14.0, 26.0)	24.0 (19.5, 34.0)	<0.001 †

^1^ Continuous variables are presented as mean (standard deviation) if normally distributed, or as median (1st quartile, 3rd quartile) otherwise. ^ excluding females consuming more than 20 g of ethanol/day and males consuming more than 30 g of ethanol/day (*) significantly different compared to the middle category (†) indicates normal distribution not met.

**Table 2:** Cross-sectional multivariable logistic regression model for the association between NAFLD presence and Mediterranean-type diet (*n* = 477) ^1^.

Variable	Odds Ratio for NAFLD Presence	95% Confidence Interval
MedDietScore (per 1/55)	0.85	(0.80, 0.91)
Age (per 1 year)	0.99	(0.97, 1.02)
Gender (male vs. female)	5.05	(2.70, 9.43)
Education level (per 1 school year)	1.00	(0.92, 1.08)
Smoking current, (yes vs. no)	0.72	(0.40, 1.31)
Physical activity (yes vs. no)	0.54	(0.29, 1.01)
C-reactive protein (per 1 mg/L)	1.28	(1.15, 1.42)

^1^ Odds ratios and 95% confidence intervals derived from logistic regression. NAFLD: Non-alcoholic fatty liver disease.

**Table 3:** Logistic regression models predicting 10-year CVD risk and the role of NAFLD and MedDietScore (*n =* 477) ^1^.

Model	Model 1 OR (95% CI)	Model 2 OR (95% CI)	Model 3 OR (95% CI)
NAFLD (yes vs. no)	2.4 (1.38, 4.29)	1.35 (0.71, 2.58)	0.93 (0.42, 2.03)
MedDietScore (per 1/55) *	0.88 (0.84, 0.92)	0.99 (0.85, 0.94)	0.87 (0.80, 0.96)

OR: Odds ratio; CI: Confidence interval; NAFLD: Non-alcoholic fatty liver disease. * MedDietScore consists of 11 questions and each question had six possible answers with scores 0–5. The theoretical total score ranges from 0 to 55 with higher scores indicating a higher level of adherence to Mediterranean diet. The odds ratios refer to each one-point increase in the MedDietScore. ^1^ *Model 1:* Univariable models. *Model 2:* MedDietScore + NAFLD. *Model 3:* Fully adjusted model (Model 2 + gender, age, age * tertiles of MedDietScore interaction, C-reactive protein, smoking status, sedentary lifestyle and education level.

## Data Availability

Data described in the manuscript, code book, and analytic code will be made available upon request.

## Abbreviations

NAFLD: non-alcoholic fatty liver disease; CVD, cardiovascular disease; FLI, fatty liver index; T2D, type 2 diabetes; IPAQ, international physical activity questionnaire; BMI, body mass index; FFQ, food frequency questionnaire; CHD, chronic heart disease; GGT, gamma-glutamyl transferase; SEM, structural equation modelling; CRP, C-reactive protein.
